# Wheat phytase potentially protects HT-29 cells from inflammatory nucleotides-induced cytotoxicity

**DOI:** 10.5713/ab.23.0031

**Published:** 2023-06-26

**Authors:** Jeongmin An, Jaiesoon Cho

**Affiliations:** 1 Department of Animal Science and Technology, Konkuk University, Seoul 05029, Korea

**Keywords:** Cytotoxicity, HT-29 Cell, Inflammatory Nucleotides, Wheat Phytase

## Abstract

**Objective:**

The aim of this study was to investigate the protective effect of wheat phytase as a structural decomposer of inflammatory nucleotides, extracellular adenosine triphosphate (ATP), and uridine diphosphate (UDP) on HT-29 cells.

**Methods:**

Phosphatase activities of wheat phytase against ATP and UDP was investigated in the presence or absence of inhibitors such as L-phenylalanine and L-homoarginine using a Pi Color Lock gold phosphate detection kit. Viability of HT-29 cells exposed to intact- or dephosphorylated-nucleotides was analyzed with an EZ-CYTOX kit. Secretion levels of pro-inflammatory cytokines (IL-6 and IL-8) in HT-29 cells exposed to substrate treated with or without wheat phytase were measured with enzyme-linked immunosorbent assay kits. Activation of caspase-3 in HT-29 cells treated with intact ATP or dephosphorylated-ATP was investigated using a colorimetric assay kit.

**Results:**

Wheat phytase dephosphorylated both nucleotides, ATP and UDP, in a dose-dependent manner. Regardless of the presence or absence of enzyme inhibitors (L-phenylalanine and L-homoarginine), wheat phytase dephosphorylated UDP. Only L-phenylalanine inhibited the dephosphorylation of ATP by wheat phytase. However, the level of inhibition was less than 10%. Wheat phytase significantly enhanced the viability of HT-29 cells against ATP- and UDP-induced cytotoxicity. Interleukin (IL)-8 released from HT-29 cells with nucleotides dephosphorylated by wheat phytase was higher than that released from HT-29 cells with intact nucleotides. Moreover, the release of IL-6 was strongly induced from HT-29 cells with UDP dephosphorylated by wheat phytase. HT-29 cells with ATP degraded by wheat phytase showed significantly (13%) lower activity of caspase-3 than HT-29 cells with intact ATP.

**Conclusion:**

Wheat phytase can be a candidate for veterinary medicine to prevent cell death in animals. In this context, wheat phytase beyond its nutritional aspects might be a novel and promising tool for promoting growth and function of intestinal epithelial cells under luminal ATP and UDP surge in the gut.

## INTRODUCTION

Extracellular nucleotides such as adenosine triphosphate (ATP) and uridine diphosphate (UDP) are important biological molecules essential to all living organisms. They are critical components in both intracellular and extracellular signals [1]. In general, ATP plays an important role as an essential energy source for various biological mechanisms such as metabolism and respiration in enzymatic reactions of most organisms [2,3]. UDP, a key factor of glycogenesis, is a pyrimidine nucleotide intermediator that is crucial for *de novo* biosynthesis of nucleic acid [4]. These two nucleotides are found in almost all biological reactions, including immune responses [5,6].

At first glance, ATP and UDP seem to only have positive effects on living organisms based on roles of ATP and UDP mentioned above. However, extracellular nucleotides and their sugar conjugate forms such as UDP-glucose and UDP-galactose can also act as danger-associated molecular patterns (DAMPs) secreted from incurred cells [7]. Specifically, extracellular ATP works as a DAMP in human cells when ATP levels exceed the capacity of extracellular ATPases [8,9]. An increase in extracellular ATP might have been induced by bacterial infection as release of ATP has been reported during growth of Gram-negative and Gram-positive bacteria such as *Acinetobacter junii*, *Pseudomonas aeruginosa*, *Klebsiella pneumoniae*, *Klebsiella oxytoca*, and *Staphylococcus aureus* [3]. Ultimately, excessive increases of extracellular ATP and UDP during bacterial infection can induce caspase-dependent apoptosis that can destroy various animal cells including thymocytes, hepatocytes, microglial, myeloid cells, and hippocampal organotypic cells [10–12]. Previous studies have succeeded in elucidating immunologically detrimental aspects of extracellular ATP by demonstrating that extracellular ATP can induce apoptosis and neuronal dysfunction [13]. In addition, according to previous studies [12,14], not only ATP but also uracil nucleotides (UTP and UDP) can induce apoptosis of astrocytes by inhibiting cell proliferation and increasing NO production in microglia. ATP is also needed for endotoxemia to trigger systemic inflammation [15]. Cauwels et al [15] have reported that removal of systemic extracellular ATP can dampen toxicity and damage in systemic inflammatory response syndrome in a murine model. Dephosphorylation of ATP can alleviate apoptosis, cellular disintegration, mitochondrial damage, and intestinal breakdown [11]. Likewise, degradation of extracellular nucleotides by enzymatic dephosphorylation may reduce their activity such as apoptosis induction and cell damage. However, direct effects of nucleotides and enzymatically dephosphorylated nucleotides on animal cells have not been elucidated yet.

Wheat phytase is well known as a nutritional feed additive for improving phosphate availability of monogastric animals. It can also reduce phosphate pollution in livestock husbandry [16]. Interestingly, wheat phytase classified as multiple inositol polyphosphate phosphatase (MINPP) can hydrolyze ATP and non-specifically dephosphorylate p-nitrophenyl phosphate, 2,3-bisphosphoglyceric acid (2,3-BPG), and diphospho-*myo*-inositol pentakisphosphate (PP-InsP5) [16,17]. However, the progress of research on the enzymatic ability of wheat phytase to affect immunity and viability of living cells by dephosphorylating inflammatory nucleotides has been quite slow [6,18]. The aim of this study was to investigate the protective effect of wheat phytase as a structural decomposer of inflammatory nucleotides, extracellular ATP and UDP, on HT-29 cells, an intestinal epithelial cell line.

## MATERIALS AND METHODS

### Reagents and maintenance of cell culture

Nucleotides (ATP and UDP) and wheat phytase used in this study were purchased from Sigma-Aldrich (St. Louis, MO, USA). Wheat phytase was reconstituted in endotoxin-free water (Sigma-Aldrich, USA) and residual inorganic phosphate was removed through Pi-bond resin (Innova Biosciences, Cambridge, UK). A malachite green-based Pi Color Lock gold phosphate detection kit was procured from Innova Biosciences (UK). L-phenylalanine and L-homoarginine as inhibitors of dephosphorylation, were procured from Sigma-Aldrich (USA). For cell viability assay, an EZ-CYTOX kit was purchased from DogenBio (Seoul, Korea). Cymax human interleukin-6 (IL-6) and IL-8 enzyme-linked immunosorbent assay (ELISA) kits used for IL-6 and IL-8 secretion assay were obtained from Ab FRONTIER (Seoul, Korea). A caspase-3/CPP32 colorimetric assay kit used to measure the activity of caspase-3, marker of programmed cell death, was sourced from BioVision (Milpitas, CA, USA). Human colorectal adenocarcinoma cell line, HT-29, was obtained from ATCC (Manassas, VA, USA). Cells were cultured in McCoy’s 5A medium purchased from Gibco Life technologies (Carlsbad, CA, USA). The medium was supplemented with 10% fetal bovine serum and 1% penicillin-streptomycin solution, both of which were purchased from Gibco Life technologies (USA). These cells were cultured at 37°C in a humidified air incubator with 5% CO_2_.

### Dephosphorylation assay for nucleotides

The activity of wheat phytase as a phosphatase against ATP and UDP was determined at 37°C for 15 min. ATP or UDP (100 μM) was treated with different concentrations (0.0715, 0.143, and 0.286 mU/mL) of wheat phytase in 50 mM sodium-acetate buffer (pH 5.0). Phosphatase activities of wheat phytase (0.143 mU/mL) against ATP and UDP was measured with or without phosphatase inhibitors such as L-phenylalanine and L-homoarginine (10 mM). Amounts of free orthophosphate released from nucleotides were measured at optical density (OD) 635 nm using a Pi Color Lock gold kit (Innova Biosciences, UK) based on malachite green detection according to the manufacturer’s instructions.

### Cell-viability assay

HT-29 cells were seeded onto 96-well plates at a density of 10^4^ cells/well and cultured until confluency of each well reached 80%. Nucleotides (12 mM of ATP and UDP) were pretreated at 37°C for 1 h with or without wheat phytase (286 mU/mL) in distilled water (E-Toxate Water; Sigma-Aldrich, USA). Then 10 μL of aliquot was taken from the reaction mixture and applied to cells in each well. After 24 h of incubation, 10 μL of EZ-CYTOX was used to treat cells in each well. Cell viabilities were then measured at OD 450 nm using the Synergy 2 microplate reader (BioTek, Winooski, VT, USA) according to the manufacturer’s instructions.

### Interleukin-6 and -8 assay

This assay was started by seeding HT-29 cells (10^4^ cells/well) onto 96-well plate. Cells were cultured until confluence of each well reached 80%. Samples used to measure IL-8 and IL-6 secretion from HT-29 cells were pretreated as follow: ATP (12 mM) or UDP (1 mM) was incubated with wheat phytase (1.43 U/mL) at 37°C for 1 h. Then, 10 μL of the reaction mixture was added to HT-29 cells. After 24 h incubation, the cell culture medium containing IL-8 and IL-6 released from cells was analyzed with ELISA kits and OD 450 nm was measured according to the manufacturer’s protocols.

### Caspase-3/CPP32 assay

First, HT-29 cells used for caspase-3/CPP32 assay were seeded onto 6-well plate at a density of 3×10^5^ cells/well and cultured until cells covered 80% of each well. ATP (12 mM) was dephosphorylated by wheat phytase (286 mU/mL) at 37°C for 1 h. Enzyme-treated ATP was then added to the plate containing HT-29 cells. After incubation for 24 h, OD 405 nm of each sample was measured according to instructions provided by the Caspase-3/CPP32 colorimetric assay kit by the manufacturer.

### Statistical analysis

Statistical significance among and between groups was determined by one-way analysis of variance using the general linear model function of SAS 9.4 (SAS Institute, Cary, NC, USA) followed by Duncan’s multiple range test and by Student’s *t*-test, respectively. Statistical significance is defined when p-values are less than 0.05.

## RESULTS

### Dephosphorylation of ATP and UDP by wheat phytase

**Figure 1:**
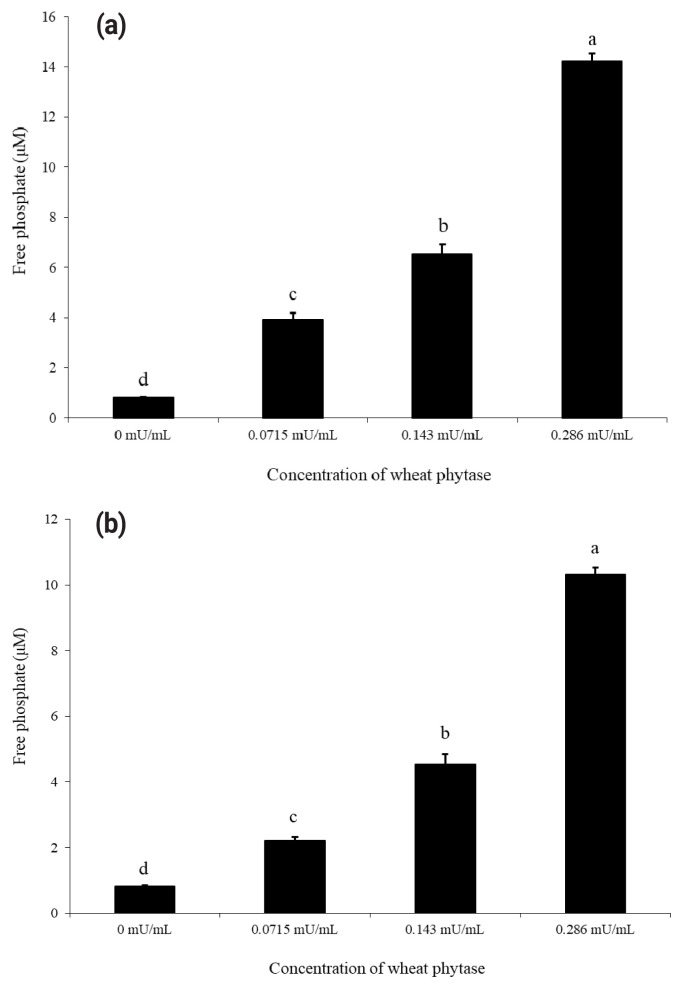
Phosphatase activities of wheat phytase towards adenosine triphosphate (ATP) (a) and uridine diphosphate (UDP) (b) using different amounts of enzyme. Data are expressed as mean and standard errors from three experiments. ^a–d^ Means lacking common superscripts differ significantly (p<0.05).

**Figure 2:**
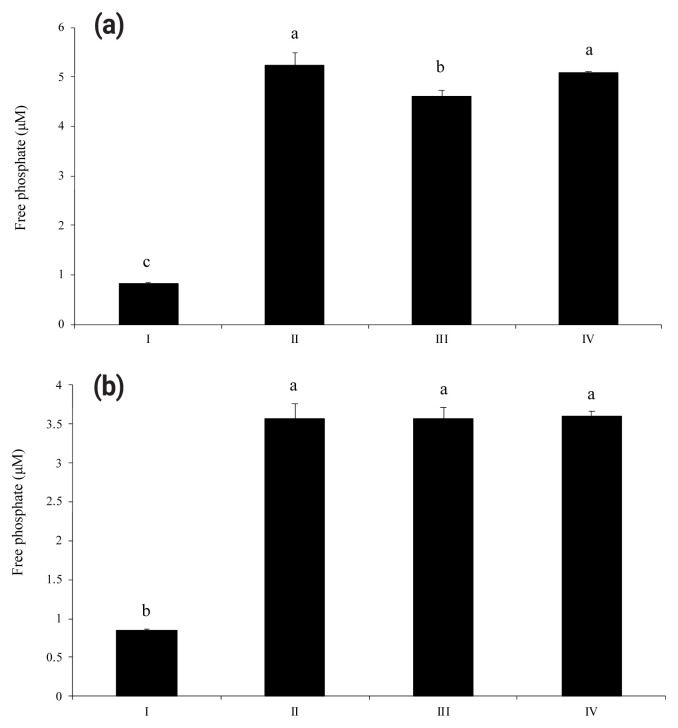
Phosphatase activities of wheat phytase towards adenosine triphosphate (ATP) (a) and uridine diphosphate (UDP) (b) in the presence of L-phenylalanine or L-homoarginine. I, intact substrate; II, enzyme treatment; III, enzyme treatment in the presence of L-phenylalanine; IV, enzyme treatment in the presence of L-homoarginine. Data are expressed as mean and standard errors from three experiments. ^a–c^ Means lacking common superscripts differ significantly (p<0.05).

As shown in Figure 1(a) and 1(b), wheat phytase dephosphorylated both nucleotides, ATP and UDP, dose-dependently. The highest levels of free phosphate, 14.2 μM and 10.3 μM, were separated from ATP and UDP, respectively, at wheat phytase concentration of 0.286 mU/mL. Wheat phytase was more effective in dephosphorylating ATP than UDP. Dephosphorylation of UDP by wheat phytase was unaffected by L-phenylalanine, a representative tissue-specific inhibitor, or L-homoarginine, a tissue-non-specific alkaline phosphatase inhibitor (Figure 2b). L-homoarginine did not affect the catalytic property of wheat phytase to degrade ATP either (Figure 2a). Although wheat phytase-induced degradation of ATP was inhibited by L-phenylalanine, the level of inhibition was less than 10%.

### ATP- and UDP-induced cell death

**Figure 3:**
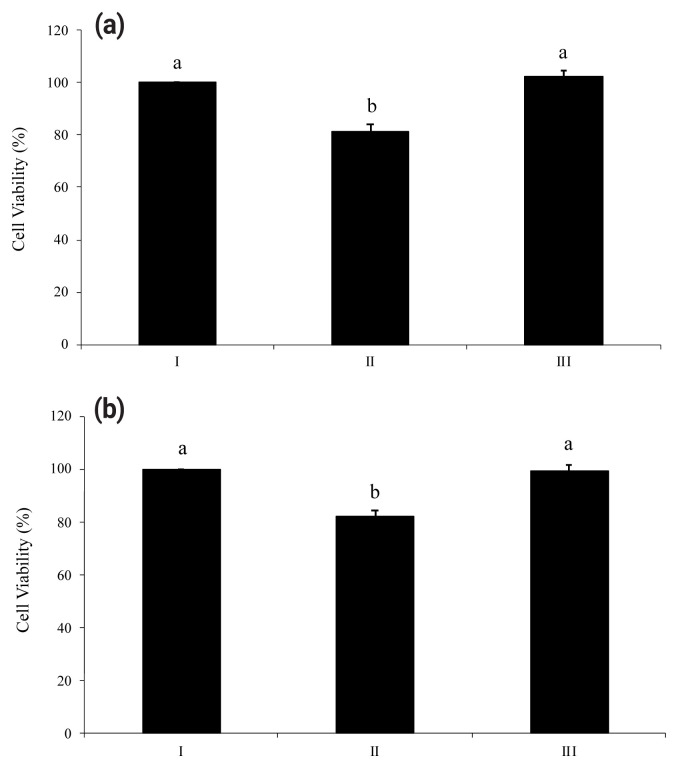
Viabilities of HT-29 cells exposed to adenosine triphosphate (ATP) (a) and uridine diphosphate (UDP) (b) hydrolyzed by wheat phytase. I, no addition; II, intact substrate; III, substrate treated with wheat phytase. Data are expressed as mean and standard errors from three experiments. ^a,b^ Means lacking common superscripts differ significantly (p<0.05).

Proinflammatory nucleotides ATP and UDP significantly induced death of HT-29 cells. As shown in Figure 3(a) and Figure 3(b), viabilities of HT-29 cells treated with ATP or UDP were 18% lower than a non-treated group. However, both figures (Figure 3a and 3b) showed the ability of wheat phytase to successfully increase the viability of substrate-treated HT-29 cells. There was no significant difference in viability between the group in which enzyme-treated nucleotides were added to HT-29 cells and the group in which cells were not treated.

### Effects of wheat phytase-treated ATP and UDP on IL-8 and IL-6 release in HT-29 cells

**Figure 4:**
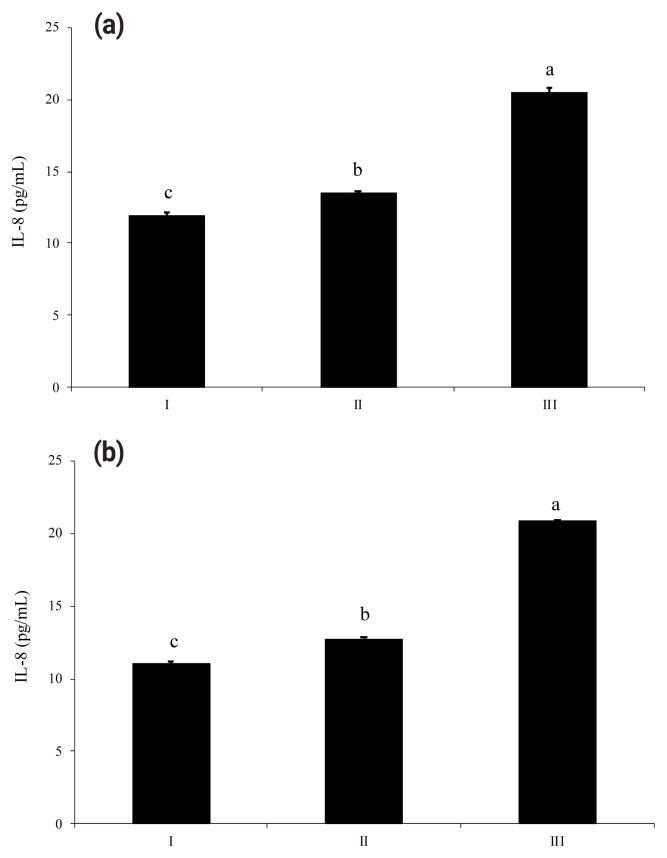
Effects of adenosine triphosphate (ATP) (a) and uridine diphosphate (UDP) (b) hydrolyzed by wheat phytase on interleukin-8 (IL-8) release from HT-29 cells. I, no addition; II, intact substrate, III, substrate treated with wheat phytase. Data are expressed as mean and standard errors from three experiments. ^a–c^ Means lacking common superscripts differ significantly (p<0.05).

**Figure 5:**
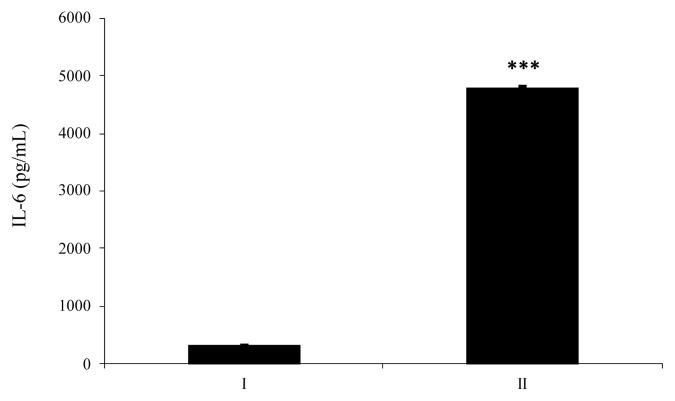
Effect of uridine diphosphate (UDP) hydrolyzed by wheat phytase on interleukin-6 (IL-6) release from HT-29. I, intact substrate; II, substrate treated with wheat phytase. Data are expressed as mean and standard errors from three experiments (*** p<0.001; Student’s *t*-test).

HT-29 cells supplemented with enzyme-treated ATP showed increased secretion of IL-8 by approximately 152% compared to cells supplemented with intact ATP (Figure 4a). As shown in Figure 4(b), IL-8 secreted from HT-29 cells treated with dephosphorylated-UDP was 1.6-fold higher than that secreted from cells treated with an intact substrate. In the case of IL-6 secretion from HT-29 cells, wheat phytase-treated UDP strongly increased the level of IL-6 secretion compared to intact UDP, with an increase of almost 14.5-fold (Figure 5).

### Activation of caspase-3, a marker of apoptosis, alleviated by wheat phytase

**Figure 6:**
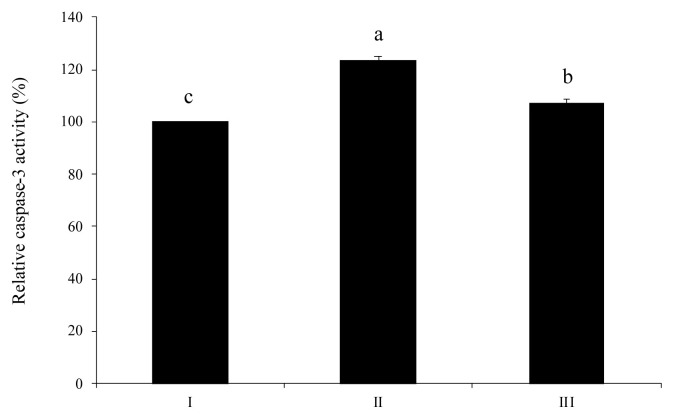
Caspase-3 activation in HT-29 cells exposed to adenosine triphosphate (ATP) hydrolyzed by wheat phytase. I, no addition; II, intact substrate; III, substrate treated with wheat phytase. Data are expressed as mean and standard errors from three experiments. ^a–c^ Means lacking common superscripts differ significantly (p<0.05).

Activation of caspase-3 stimulated by extracellular ATP was alleviated by wheat phytase (Figure 6). As a result, the level of caspase-3 in HT-29 cells treated with an intact ATP was increased 1.2-fold more than that in untreated control cells. When HT-29 cells were treated with dephosphorylated ATP, the activation level of caspase-3 was 13% lower than that in cells treated with pure ATP.

## DISCUSSION

We aimed to investigate the protective effect of wheat phytase as a structural decomposer of inflammatory nucleotides, extracellular ATP, and UDP on HT-29 cells in the present study. While physiologically normal concentration of plasma ATP is between 400 to 700 nM, intestinal luminal ATP concentration is relatively higher (1 to 10 mM) because ATP is secreted from intestinal necrotic cells and bacteria [2,19]. Luminal ATP levels are regulated by ecto-ATPase, an endogenous phosphatase in intestinal epithelial cells [20]. However, ecto-ATPase is known to be less efficient than intestinal alkaline phosphatase (IAP) in ATP hydrolysis. The function of IAP can be blocked under abnormal physiological conditions such as fasting [19]. When the concentration of extracellular ATP exceeds the capacity of ATPase, ATP starts to act as a DAMP [9]. Moreover, since ATP is required as an energy source during apoptosis, regulating ATP levels is important [11]. Indeed, recent studies have shown that ATP level is related to neuron dysfunction as ATP level can determine cell death mode of human leukemia cells (HL-60 cells) [8,13]. Thus, regulation of ATP levels is a critical factor in preventing cellular disintegration, apoptosis, intestinal barrier disruption, and even mortality [19]. Although ecto-ATPase and IAP in intestinal epithelial cells are known to contribute to lumen ATP regulation and reduction of inflammatory responses, respectively, associations between extracellular ATP and intestinal epithelial cells are unknown [20].

Like ATP, uracil nucleotides (i.e., UTP and UDP) can inhibit cell proliferation and increase apoptosis of astrocyte [12,21]. Apoptosis is associated with the production of reactive nitrogen species such as nitric oxide (NO). The production of NO is triggered by UDP converted from UTP [12,14,21]. In addition, uracil nucleotides conjugated to sugar such as UDP-glucose and UDP-galactose can act as DAMPs in cells [7]. Taken together, both ATP and UDP can induce apoptosis in certain environments and act as DAMPs [22]. In this respect, lowering levels of ATP and UDP through dephosphorylation might affect immune responses including proliferation and survival of intestinal epithelial cells.

Interestingly, in this research, wheat phytase, a novel phytogenic phosphatase, acted as an effective ATP degrader in HT-29 cells just like IAP and ecto-ATPase. L-homoarginine, a tissue-non specific alkaline phosphatase inhibitor, did not inhibit phosphatase activity of wheat phytase at all. However, L-phenylalanine, a representative tissue-specific phosphatase inhibitor, inhibited wheat phytase, although the inhibition level was less than 10%. Extracellular ATP reduced cell viability of HT-29 by about 20%, similarly to results obtained from a human cervical cancer cell line, SiHa [23]. However, dephosphorylation of extracellular ATP by wheat phytase significantly enhanced the cell viability of HT-29 and increased IL-8 release from HT-29 cells compared to intact ATP. This result shows that dephosphorylation of ATP by wheat phytase can protect HT-29 cells from ATP-induced damage. Likewise, a previous study has reported that astrocytes could resist ATP-induced cell death because they have high rates of ATP hydrolysis under physiological conditions [11]. Elevated IL-6 or IL-8 secretion is associated with proliferation, angiogenesis, and survival of many cancer cell lines [24–27]. It is mainly mediated by the activation of P2-purinergic receptors such as G-protein-coupled receptors P2Y and ligand-gated ion channels P2X [25]. Meanwhile, reactive hydrolysates such as ADP and adenosine after ATP digestion of wheat phytase can readily induce the expression of functional receptors in HT-29 cells. These receptors are implicated in cell growth, differentiation, and IL-8 secretion [24]. Similar to the study on human gastric carcinoma cells exposed to ATP [13], this study also showed that the reduction in cell viability of HT-29 induced by extracellular ATP was associated with caspase-3 dependent apoptosis. As shown in results of the present study, HT-29 cells treated with wheat phytase-degraded ATP had 13% lower activity of caspase-3, a distinct death proteases frequently activated in apoptosis of mammalian cells, than cells treated with intact ATP. This indicates that dephosphorylation of ATP by wheat phytase could be a new strategy to enhance the viability of HT-29 cells and mitigate ATP-induced caspase-3-dependent apoptosis [11].

Consistent with trends shown in other previous studies [14,28], UDP was dephosphorylated by wheat phytase in a dose-dependent manner in the present study. The dephosphorylation effect of the enzyme was maintained even when inhibitors such as L-phenylalanine and L-homoarginine were added. Wheat phytase restored the viability of HT-29 cells through dephosphorylation of UDP. In other words, wheat phytase protected HT-29 cells from UDP-induced damage. Wheat phytase-treated UDP promoted the secretion of cytokines, IL-6 and IL-8, from HT-29 cells. IL-6 and IL-8 are multifunctional cytokines that play central role in host protection due to their wide ranges of immune activities [29]. These results showed that wheat phytase could enhance cell viability of HT-29 against UDP-induced cytotoxicity by upregulating the release of both IL-6 and IL-8 known to be associated with proliferation and survival of many cells [24, 25]. The present study also focuses on the possibility that wheat phytase may function as a direct up-regulator of IL-6 or IL-8 expression like HSP60 (heat shock protein 60) to enhance the cell survival against ATP or UDP stimulus [27], which is a new approach different from our previous studies that the enzyme play a role in decreasing the IL-8 release for cell viability against phosphorylated substrates such as LPS (lipopolysaccharide) and inorganic polyphosphates, mainly derived from exogenous pathogenic bacteria [30,31].

Until now, for improving gut health, studies have mainly focused on modulating the number and composition of intestinal microbiota using probiotics and prebiotics [32]. Considering that impairment and malfunction of intestinal epithelial cells can aggravate intestinal inflammation [33], intestinal epithelial cells are also key factors in maintaining a healthy relationship between intestinal microbiota and host immunity [33]. In a previous report, nucleotides increased apoptosis cells in the jejunum and ileum of weaned piglets [34]. Thus, protecting the viability of intestinal epithelial cells from extracellular nucleotides in the intestine has a great impact on overall productivity as well as intestinal health of living organisms in animal husbandry. In this study, ATP- and UDP-mediated death of intestinal epithelial cells was potentially alleviated by wheat phytase, implying that the enzyme could be a candidate for improving gut health in animals. Furthermore, this research proposes that wheat phytase can act as a bifunctional agent. It can be considered as a feed additive to improve the production performance. It can also be used as a candidate for veterinary medicine to prevent cell death in animals. In this context, wheat phytase beyond its nutritional aspects might be a novel and promising tool for promoting the growth and function of intestinal epithelial cells under luminal ATP and UDP surge in the gut. As a future study, it will be interesting to reveal how procaspase-3, the inactive precursor zymogen of caspase-3, may function as a regulator of caspase-3 activity in HT-29 cells exposed to intact ATP and wheat phytase-treated ATP, even though the relationship between overexpression of procaspase-3 and insufficient caspase-3 activity is puzzling in various cancer cell lines until now [35], and the mechanism by which procaspase-3 is converted into active caspase-3 remains unclear [36].

## References

[1] Ng S, Lim HS, Ma Q, Gao Z (2016). Optical aptasensors for adenosine triphosphate. Theranostics.

[2] Bours MJL, Swennen ELR, Di Virgilio F, Cronstein BN, Dagnelie PC (2006). Adenosine 5′-triphosphate and adenosine as endogenous signaling molecules in immunity and inflammation. Pharmacol Ther.

[3] Mempin R, Tran H, Chen C, Gong H, Ho KK, Lu S (2013). Release of extracellular ATP by bacteria during growth. BMC Microbiol.

[4] Lecca D, Ceruti S (2008). Uracil nucleotides: from metabolic intermediates to neuroprotection and neuroinflammation. Biochem Pharmacol.

[5] Burnstock G (2007). Physiology and pathophysiology of purinergic neurotransmission. Physiol Rev.

[6] Trautmann A (2009). Extracellular ATP in the immune system: more than just a “danger signal”. Sci Signal.

[7] Brautigam VM, Dubyak GR, Crain JM, Watters JJ (2008). The inflammatory effects of UDP-glucose in N9 microglia are not mediated by P2Y14 receptor activation. Purinergic Signal.

[8] Grusch M, Polgar D, Gfatter S (2002). Maintenance of ATP favours apoptosis over necrosis triggered by benzamide riboside. Cell Death Differ.

[9] Hasan D, Satalin J, Van der Zee P (2018). Excessive extracellular ATP desensitizes P2Y2 and P2X4 ATP receptors provoking surfactant impairment ending in ventilation-induced lung injury. Int J Mol Sci.

[10] Marques-da-Silva C, Chaves MM, Chaves SP (2011). Infection with Leishmania amazonensis upregulates purinergic receptor expression and induces host-cell susceptibility to UTP-mediated apoptosis. Cell Microbiol.

[11] Morrone FB, Horn AP, Stella J (2005). Increased resistance of glioma cell lines to extracellular ATP cytotoxicity. J Neurooncol.

[12] Quintas C, Pinho D, Pereira C (2014). Microglia P2Y6 receptors mediate nitric oxide release and astrocyte apoptosis. J Neuroinflammation.

[13] Wang M, Ren L (2006). Growth inhibitory effect and apoptosis induced by extracellular ATP and adenosine on human gastric carcinoma cells: involvement of intracellular uptake of adenosine. Acta Pharmacol Sin.

[14] Moss AK, Hamarneh SR, Mohamed MMR (2013). Intestinal alkaline phosphatase inhibits the proinflammatory nucleotide uridine diphosphate. Am J Physiol Gastrointest Liver Physiol.

[15] Cauwels A, Rogge E, Vandendriessche B, Shiva S, Brouckaert P (2014). Extracellular ATP drives systemic inflammation, tissue damage and mortality. Cell Death Dis.

[16] Dionisio G, Holm PB, Brinch-Pedersen H (2007). Wheat (Triticum aestivum L.) and barley (Hordeum vulgare L.) multiple inositol polyphosphate phosphatases (MINPPs) are phytases expressed during grain filling and germination. Plant Biotechnol J.

[17] Kilaparty SP, Singh A, Baltosser WH, Ali N (2014). Computational analysis reveals a successive adaptation of multiple inositol polyphosphate phosphatase 1 in higher organisms through evolution. Evol Bioinform.

[18] Di Virgilio F, Boeynaems JM, Robson SC (2009). Extracellular nucleotides as negative modulators of immunity. Curr Opin Pharmacol.

[19] Malo MS, Moaven O, Muhammad N (2014). Intestinal alkaline phosphatase promotes gut bacterial growth by reducing the concentration of luminal nucleotide triphosphates. Am J Physiol Gastrointest Liver Physiol.

[20] Lallès JP (2014). Luminal ATP: the missing link between intestinal alkaline phosphatase, the gut microbiota, and inflammation?. Am J Physiol Gastrointest Liver Physiol.

[21] Grbic DM, Degagné É, Langlois C, Dupuis AA, Gendron FP (2008). Intestinal inflammation increases the expression of the P2Y6 receptor on epithelial cells and the release of CXC chemokine ligand 8 by UDP. J Immunol.

[22] Azroyan A, Cortez-Retamozo V, Bouley R (2015). Renal intercalated cells sense and mediate inflammation via the P2Y14 receptor. PloS one.

[23] Mello PeA, Filippi-Chiela EC, Nascimento J (2014). Adenosine uptake is the major effector of extracellular ATP toxicity in human cervical cancer cells. Mol Biol Cell.

[24] Bahrami F, Kukulski F, Lecka J (2014). Purine-metabolizing ectoenzymes control IL-8 production in human colon HT-29 cells. Mediators Inflamm.

[25] Braganhol E, Kukulski F, Lévesque SA (2015). Nucleotide receptors control IL-8/CXCL8 and MCP-1/CCL2 secretions as well as proliferation in human glioma cells. Biochim Biophys Acta.

[26] Chen X, Wei J, Li C (2018). Blocking interleukin-6 signaling inhibits cell viability/proliferation, glycolysis, and colony forming activity of human medulloblastoma cells. Int J Oncol.

[27] Kumar S, O’Malley J, Chaudhary AK (2019). Hsp60 and IL-8 axis promotes apoptosis resistance in cancer. Br J Cancer.

[28] Lei W, Ni H, Herington J, Reese J, Paria BC (2015). Alkaline phosphatase protects lipopolysaccharide-induced early pregnancy defects in mice. PLoS One.

[29] Simpson RJ, Hammacher A, Smith DK, Matthews JM, Ward LD (1997). Interleukin-6: structure-function relationships. Protein Sci.

[30] An J, Cho J (2021). Wheat phytase can alleviate the cellular toxic and inflammatory effects of lipopolysaccharide. J Anim Sci Technol.

[31] An J, Cho J (2022). Effect of long-chain inorganic polyphosphate treated with wheat phytase on interleukin 8 signaling in HT-29 cells. Anim Biosci.

[32] Callaway TR, Edrington TS, Harvey RB, Anderson RC, Nisbet DJ (2012). Prebiotics in food animals, a potential to reduce foodborne pathogens and disease. Rom Biotechnol Lett.

[33] Okumura R, Takeda K (2017). Roles of intestinal epithelial cells in the maintenance of gut homeostasis. Exp Mol Med.

[34] Valini GAC, Duarte MS, Calderano AA (2021). Dietary nucleotide supplementation as an alternative to in-feed antibiotics in weaned piglets. Animal.

[35] Boudreau MW, Peh J, Hergenrother PJ (2019). Procaspase-3 overexpression in cancer: a paradoxical observation with therapeutic potential. ACS Chem Biol.

[36] Ponder KG, Boise LH (2019). The prodomain of caspase-3 regulates its own removal and caspase activation. Cell Death Discov.

